# Assessing malaria control in the Kassena-Nankana district of northern Ghana through repeated surveys using the RBM tools

**DOI:** 10.1186/1475-2875-6-103

**Published:** 2007-08-04

**Authors:** Seth Owusu-Agyei, Elizabeth Awini, Francis Anto, Thomas Mensah-Afful, Martin Adjuik, Abraham Hodgson, Edwin Afari, Fred Binka

**Affiliations:** 1 Kintampo Health Research Centre, P.O. Box 200, Kintampo, Ghana; 2 Navrongo Health Research Centre, Navrongo, P.O. Box 114, Navrongo, Ghana; 3 Kassena-Nankana District Health Management Team, Navrongo, Ghana; 4 School of Public Health, University of Ghana, Legon, Ghana

## Abstract

**Background:**

The goal of Roll Back Malaria (RBM) is to reduce malaria morbidity and mortality by 50% by the year 2010, and still further thereafter until the disease becomes no more a threat to public health. To contribute to the monitoring and evaluation process of this goal, two surveys were carried out in 2000 and 2003 in households and health facilities in the Kassena-Nankana district, northern Ghana using the RBM-WHO/AFRO monitoring and evaluation tools for malaria control activities.

**Methods:**

Data were collected from mothers/caretakers on signs/symptoms of the most recent malaria attack for their under five year old children; the management actions that they took and their perception of health services provided at the health facilities, bednet use, antenatal attendance and place of delivery for the most recent pregnancy, malaria prophylaxis during their last pregnancy. Community health workers and herbalist/traditional healers were also interviewed about the types of health services they provide to community members.

**Results:**

The results revealed a significant improvement in knowledge among mothers/caretakers over the three-year period; this affected caretakers' initial management of illnesses of their young children. The management in terms of the type and dosage of drugs used also improved significantly (p < 0.0001) over the period. Reported insecticide-treated bed net use among children under-five years and pregnant women significantly increased between 2000 and 2003 (p < 0.0001). Health professionals had improved on adoption of their quality of care roles.

The intensification of malaria control activities and awareness creation in this district over a three year period had started demonstrating positive results towards reducing malaria disease burden.

**Conclusion:**

Periodic performance assessments through surveys as described and prompt feedback of results to stakeholders in the locality serves as a catalyst to improving malaria control in malaria-endemic countries.

## Background

Malaria is the world's most important tropical parasitic disease; killing more people than any other communicable disease, except tuberculosis. Prevention is the best protection from malaria. It includes individual protection, such as the use of insecticide-treated bed nets, mosquito repellants and drug prophylaxis for pregnant women; community measures, such as the control of mosquito breading sites, insecticide spraying and drainage.

Morbidity and mortality are particularly high among pregnant women, young children, and sick persons lacking immunity. Malaria during pregnancy causes severe anaemia and low birth weight at delivery as well as contributes to maternal deaths in malaria-endemic areas. Present estimates are that around 350–500 million clinical disease episodes occur annually [1]. Around 60% of the cases of clinical malaria and over 80% of the deaths occur in Africa south of the Sahara. Most of the over one million Africans who die from malaria each year are children under five years of age [2].

In Ghana, malaria accounts for more than 44% of reported outpatient visits and an estimated 22% of under-five mortality. Reported malaria cases represent only a small proportion of the actual number of episodes as majority of people with symptomatic infections are treated at home and are, therefore, not reported [1].

In 1998, the Director General of the World Health Organization launched a global partnership, the Roll Back Malaria (RBM) initiative. The main objective of RBM is to reduce malaria morbidity and mortality by half by the year 2010 [3]; with a further reduction in morbidity and mortality of 50% and 75%, respectively, by 2015 over the achievements in 2010, until malaria ceases to be of public health importance.

Several initiatives have been undertaken in Ghana following the launching of the RBM partnership. The Ghana partnership emphasizes strengthening health services in general and making effective prevention and treatment strategies more widely available and promptly. Ghana's RBM strategy involves multi-sectoral and inter-sectoral partnerships working towards reducing death and illness caused by malaria by 50% by 2010. Some progress has been made in improving access to prompt and effective treatment, supply of insecticide-treated nets (ITNs) and using intermittent preventive treatment with sulphadoxine-pyrimethamine (SP) in pregnancy (IPT_p_). Based on evidence from drug efficacy studies, Ghana has recently changed from chloroquine to artesunate plus amodiaquine for the treatment of uncomplicated malaria. Several collaborative ITN campaigns are ongoing with RBM partners including WHO and UNICEF.

The survey benefited tremendously from an ongoing demographic surveillance system, the Navrongo demographic surveillance system, NDSS[4] that provides precise estimates of under-five mortality and malaria-specific mortality for planning of malaria control activities in the district. Malaria deaths in the district are provided through deaths recorded in the health facilities, as well as verbal autopsy reports. The NDSS routinely documents all births and deaths that occur in the district that are subsequently subjected to a verbal autopsy interview, later coded by physicians who assign the probable cause of death. RBM monitoring and evaluation tools [3] were used, backed by the NDSS to monitor progress in achievements towards the RBM goals in the Kassena-Nankana district of Ghana.

## Methods

### Study site

The surveys were carried out in the Kassena-Nankana district (KND), one of the 138 administrative districts in Ghana. KND, located in northern Ghana and bordering Burkina Faso is served by the Navrongo Health Research Centre, which runs the NDSS and documents demographic changes in the population of the district [4]. The district's population is estimated, from four-monthly demographic updates, as 142,000 people living in roughly 14,000 dispersed compounds; it covers an area of about 1,675 km^2^. There are two main seasons, the wet (June-October) and the dry (November-May). The average annual rainfall is 850–950 mm with an average temperature range of 18°C-45°C. The main occupation of the people is subsistence farming of predominantly millet, groundnuts and small herds of livestock.

**Figure 1:**
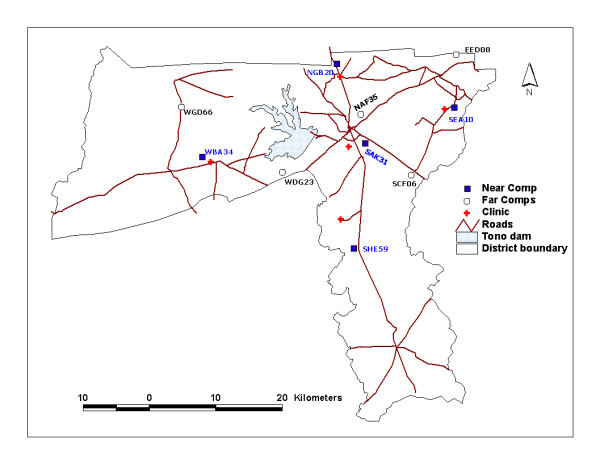
Map of Kassena-Nankana district showing location of health facilities and index compounds.

Malaria transmission is by *Anopheles gambiae *and *Anopheles funestus *and peaks at the end of the wet season [5]. The prevalence of *Plasmodium falciparum *infection among children under five years of age in the district is known to be significantly greater in the wet than in the dry season [6]. The district has one hospital and four health centres that are strategically located to serve all parts of the district (Figure 1). Each health centre serves a population of about 30,000 with the district hospital serving as a referral facility to all the health centres. Other health care providers in the district include, traditional healers, traditional birth attendants, pharmacy shop owners and itinerant drug vendors

The study covers a period when two key malaria intervention activities were ongoing in the district: 1) a district wide study of the survival benefit in treating non per os (NPO) malaria patients aged 6–71 months with rectal artesunate prior to referral to a hospital (from July 2000 to February 2004) and 2) a community-randomized trial to study the effectiveness of intermittent iron supplements and malaria chemotherapy in reducing the incidence of anaemia and clinical malaria in children as they receive EPI vaccines (from May 2000 to August 2003).

### Data collection and management

Repeated household and health facility surveys were carried out in the district during the peak to the end of the high malaria transmission season (September-November) of 2000 and 2003, using the tools developed by RBM [3]. The tools used included, community-based case study of recent illness in children under five years of age, prevention of malaria in pregnancy, availability and use of treated bed/mosquito nets and assessment of under-five mortality. The hospital tools included the checklists for malaria management/administration, out- and in-patient, laboratory and pharmacy/dispensary, as well as health care provider observation, an in-patient record review, and a checklist for management at the district health administration.

### Sampling procedure at the community level

The district was divided into five zones with reference to the five health facilities (Figure 1). Each zone was further divided into two sub-zones. Compounds within one kilometre radius from a health facility were grouped into sub-zone I and referred to as "near compounds". Compounds 10 kilometres or more away from the health facility were grouped into sub-zone II and referred to as "far compounds". In all, there were five "near sub-zones" or clusters and five "far sub-zones" or clusters. In effect, the district was divided into 10 clusters. An "index" compound was randomly selected from each of the 10 clusters using the NDSS database. From the indexed compounds, participants were recruited in a concentric manner until the required number of the various categories of respondents as recommended by the RBM monitoring-evaluation tools [2] was obtained.

### Community survey

A total of 184 (in 2000) and 185 (in 2003) mothers/caretakers with children under five years of age who were ill within 14 days preceding the surveys or were still unwell were interviewed. Data were collected on the signs and symptoms shown by the children and the type of action in teams of care that they took and within what time frame when they first noticed the children were unwell.

Data were also collected from 102 and 105 women who had delivered within six months preceding the 2000 and 2003 surveys respectively. Data collected included, parity, antenatal care, place of delivery, chemoprophylaxis during pregnancy, illness during pregnancy, and action taken at the time of illness if any. They were also interviewed on possession and use of bed nets and whether these bed nets were treated or not.

One hundred and fifteen (115) and another 104 compounds were randomly selected and surveyed respectively during the 2000 and 2003 surveys for bed net use (treated or untreated) and its availability to children under five years.

Nine Community Health Workers (CHW), selected by their communities to provide voluntary health services and 29 leaders of Community-Based Organizations (CBO) were randomly selected out of 27 and 56 respectively in the 2000 survey and interviewed on their training in basic management of ill health and participation in health related activities. Sixteen (16) CHWs and 26 CBO were proportionally selected for the 2003 survey. Of 35 medicine sellers (drug store keepers) and 45 itinerant drug vendors within the surveyed communities, 12 medicine sellers and 17 itinerant drug vendors were randomly selected in the 2000 survey and interviewed about their knowledge in recognition and management of malaria. Similarly, 15 medicine sellers and 22 itinerant drug vendors were proportionally interviewed during the 2003 survey.

Stock levels for chloroquine (CQ), the first line anti-malarial and sulphadoxine-pyrimethamine (SP), the second line anti-malarial until malaria treatment policy changed in Ghana in 2005, as well as availability of bed nets (treated or untreated) and other products such as cost for the prevention of malaria were assessed in 2000 and 2003. Information was also collected from a 28 and a 23 randomly selected traditional/spiritual healers within the surveyed communities in 2000, and 2003 respectively on the common illnesses that they attend to, how they treat malaria/fever and convulsion cases and whether they make referrals to health centres, as well as their preparedness to accept western medicine into their healing process. Finally data from the Demographic Surveillance System on all-cause mortality were extracted from the NDSS database in 2000 and 2003.

### Health facility survey

Information was collected from all the five health facilities in the district in 2000 and 2003. These included service capabilities of each health facility; antenatal services, laboratory services, child immunization and growth monitoring, illness/treatment guidelines, exemption services, and staff in-service training. Morbidity (all cause) and malaria specific outpatient records as well as admissions and deaths were reviewed. The admission records of child under five years of age as well as patients observed in consulting rooms were reviewed for appropriate management of their recent severe malaria cases. Laboratory data on the number of slides examined within the past 12 months, the proportion positive for malaria and the quality control measures put in place were also reviewed. Stock levels of chloroquine and sulphadoxine-pyrimethamine in the pharmacy/dispensaries, cost of treatment, and stock-out of anti-malarials were also carried out. Mothers were also interviewed on their assessment of the treatment for their children.

### Data analysis

Data processing and analysis was carried out using a custom-designed programme based on Epi info version 6 that was developed by Roll Back Malaria (RBM) for monitoring and evaluating malaria in the Africa region. This programme was used for the design of screens, data entry, and all the analysis. Analysis carried out included all-cause and malaria specific mortality, use of insecticide treated bed nets, malaria case management, access to effective anti-malarial drugs, chemoprophylaxis as well as treatment-seeking behaviour; both in the health facility and at home.

### Dissemination of findings

Meetings were held between the investigators and the stakeholders (including health care providers, community opinion leaders) within and outside the district immediately following on the 2000 survey where feedback on our first survey findings followed by comprehensive discussions on lapses in the malaria control activities were held. Reports were widely circulated as well. A similar process took place after the 2003 survey.

## Results

In the 2000 community survey, 67.4% of mothers/caretakers reported taking some action within the first 24 hours of their child's febrile illness i.e. getting some treatment for their under-five year old children. This improved to 93% in the 2003, a significant increase (p < 0.0001). A review by the study physicians of the type and dosage of drugs given to the children vis-à-vis the signs and symptoms described by the mothers/caretakers also revealed an improvement in the type of drugs given to the children between the 2000 and 20003 surveys (p = 0.001). However, there was no improvement in the dose regimen/administration to these children by their mothers/caretakers (Table 1). Mothers/caretakers from 'near' and 'far' compounds (near: 11% in 2000 and 8.4% in 2003) equally used herbal preparations for the treatment of their children (far: 15% in 2000 and 11.5% in 2003).

**Table 1:** Summary of treatment practices for first action (health care) taken during recent fever in children (< 5 yrs)

	*Survey period*		
	
	*2000*	*2003*	*p-value*
	
*Treatment practices*	*N = 184 *%	*N = 185 *%	
Action taken within 24 hrs	67.4	93.0	< 0.001
Appropriateness of prescription (drug prescribed vis-à-vis signs/symptoms)	39.7	56.3	0.001
Appropriateness of medication taken (dosage regimen by mother vis-à-vis national treatment guide line)	21.2	15.9	0.17

During the 2000 community survey, most mothers/caretakers reported seeking treatment for their children outside the formal health sector (Table 2), the commonest being chemical shops and drug vendors (50.5%) with only 26.6% attending government clinics. Using government health facilities however improved significantly in 2003 with 36.2% (p = 0.05) of mothers/caretakers then sending their children for treatment at those facilities as a first choice. Similarly, many more (73/185) mothers/caretakers took their sick children to the health facilities during the most recent illness in the 2003 survey than during 2000 (34/184) (p = 0.00001) (Table 3). Very few mothers/caretakers took their children to herbalists/traditional healers for treatment in both 2000 and 2003.

**Table 2:** Summary of types of treatment sought for children (< 5 years) with fever (First action taken)

	*Survey period*	
	
	*2000*	*2003*
	
*Types of treatment sought*	*(N= 184) *%	*(N= 185) *%
Herbs at home	12.0	11.4
Orthodox medicine kept at home	3.8	24.9
Chemist/Drug Vendor	50.5	24.3
Government clinic	26.6	36.2
Private clinic	0.0	0.5
Other	7.0	2.7

**Table 3:** Sources of medicines for the management of recent illness in children

	*Survey period*	
	
*Source of medicines*	*2000*	*2003*
	
	*N = 184 %*	*N = 185 %*
Herbs at Home	18.9	11.5
Clinic/Hospital	18.4	39.3
Drug Store	60.4	24.6
Left over medicine	----	23.5
Herbalist/Traditional Healer	0.9	1.1
Other	1.4	0.0

Attendance to antenatal clinic (at least once) during both surveys was found to be very high. While 96.1% (98/102) of women who delivered within six months of the 2000 survey attended antenatal clinic at least once during their most recent pregnancy, 93.3% (98/105) attended during the 2003 survey (p = 0.4). Home deliveries, reduced significantly (p = 0.01) from 71.6% in 2000 to 55.4% in 2003 (Table 4).

**Table 4:** Summary of care seeking behaviour of recently delivered pregnant women in KND

	*Survey period*		
	
	*2000*	*2003*	*p-value*
	
*Care seeking behaviour*	*(N = 102) *%	*(N = 105) *%	
	
Attended antenatal clinic	96.1	93.3	0.4
Delivered at home	71.6	55.4	0.01
Took chemoprophylaxis	47.1	21.0	< 0.001
Appropriate chemoprophylaxis	10.8	59.1	< 0.001

Of the 115 compounds surveyed in 2000, 110 (95.7%) had at least one bed net and used them the night before the survey. Of the 213 bed nets counted in the survey, 75% (159/213) had been treated with insecticide within six months prior to the survey. Only 47% of the total under-five children (241) surveyed slept under treated bed nets the night before the survey. Many more (90%) bed nets were, however, reported to have been treated within six months prior to the 2003 survey. This is significantly higher compared with the 2000 survey (p = 0.0003). A higher proportion of the under-five children 83% (130/156) surveyed in 2003 slept under treated bed nets the night prior to the survey; this is significantly higher compared with the 2000 survey (p < 0.00001).

Of the 102 nursing mothers/pregnant women interviewed in 2000, 29% slept under treated bed nets the night before the survey. Of the 110 compounds with bed nets, 92 said they did not buy the nets that they used, these were given to them free of charge. The few respondents who bought their bed nets before the 2000 survey mentioned the average cost of a bed net as $2.20 and the cost of re-treatment as $0.2. Significantly more (p = 0.00003) nursing mothers/pregnant women interviewed in 2003, (61/105) slept under treated bed nets the night before the survey than in 2000. Most (56.7%) of the 157 bed nets found were bought at a subsidised price of $0.56; these were made available by UNICEF through the District Health Management Team- [DHMT]). This time very few people (6.7%) reported having had the nets free-of-charge.

Five out of 12 drug sellers interviewed in both surveys indicated that they always offered full doses of anti-malarial drugs to customers; and that they 'prescribed' according to the national malaria treatment guidelines. The rest offered partial doses, based on what their patients could afford.

All the community health workers (CHW) affirmed in both surveys that they had received some training that enabled them to identify danger signs of severe malaria. They admitted treating patients from the community with the help of malaria treatment guidelines and health education materials. Cases deemed to be complicated were referred to the next higher level. Of those treated, 53% and 55% respectively reported with fever in the 2000 and 2003 surveys. Drugs used for treatment were usually acquired with the help of the Kassena-Nankana DHMT.

Four out of the five health facilities surveyed in 2000 indicated that they process and analyse the health data generated locally every week. The fifth health facility processed and analysed health data monthly. All the five health facilities sent their analysed information to the next higher level, but also used the information for planning at the facility level and later archive the data. By the 2003 survey all the health facilities were already processing and analysing their health data every week.

Chloroquine was the first line anti-malarial in Ghana until December 2005, Drug inventory revealed just over 3,000 tablets of chloroquine, in the government pharmacies/dispensaries compared with over 24,000 tablets in the private chemical shops/drug stores in the 2000 survey. This trend had changed significantly by the 2003 survey (Table 5). Whiles the chemical shops were stocking most of the chloroquine syrup in the 2000 survey; the situation had reversed by the time of the survey in 2003. Though SP, the second line anti-malarial drug in Ghana until December 2005 was found exclusively in the community in the 2000 survey, about equal quantities were found in the health facilities and the communities by the 2003 survey. Though four out of the five health facilities had some quinine in stock in 2000, three out of the five health facilities had some quinine in stock in 2003. Fee schedules were found in all the health facilities and anti-malarial drugs were cheapest; priced based on their cost price at the regional medical stores.

**Table 5:** Drug availability in health facilities compared with community sources

	*Health facilities*		*Communities*	
	
*Stock level*	2000	2003	2000	2003
Chloroquine Tablet	3,210	27,426	24,376	56,700
Chloroquine Syrup (L)	38.2	343.5	593.8	107.0
Chloroquine Injectable (Vials)	217	35	0	0
Sulphadoxine-pyrimetamine	0	279	1,171	1,930

Of the fifty-one mothers/caretakers interviewed after consultation and treatment in the health facilities in the 2000 survey, 66.7% admitted that the health care providers examined their children but only 33% of these children had their body temperatures checked (Table 6) and only 14 % of the caretakers were informed of the diagnosis made on their children. Recall on administration of the prescribed drugs was poor with only 33% of mothers/caretakers being able to do this correctly. Though almost all the caretakers (98.0%) were advised on how to treat the children at home, only 39% were advised to bring their children back to the health facility if they saw any danger signs of malaria later on. Most of the mothers/caretakers (92%) were, however, satisfied with the care provided to their children at the health facilities.

**Table 6:** Care provided to febrile children (probable malaria) by attending clinician at health facilities.

	*Survey period*	
	
	2000	2003
	
*Care provided*	(N = 51) %	(N = 71) %
Child examined	66.7	97.2
Child's temperature taken	33.3	49.3
Caretaker told of what child was suffering from	13.8	2.8
*Caretaker recalled correct administration of medication	33.3	87.9
Caretaker advised on how to treat child at home	98.0	84.5
Caretaker advised to return if danger signs are seen	39.2	8.5
Caretaker satisfied with care	92.2	88.7
**Appropriateness of care	0.0	0.0

* Correct administration as per national treatment guideline

** Combination of all the seven indicators

Many more (97.2%) children were examined by the attending clinician in the 2003 survey than in 2000 (p < 0.00001). The difference between the percentage of children whose body temperatures were checked in 2000 (33%) and 2003 (49%) was not statistically significant (p = 0.07). Only 3 % of the caretakers were informed of the diagnosis made on their children in the 2003 survey (Table 6). Recall on administration of the prescribed drugs was this time good with close to 88% of mothers/caretakers being able to do this correctly (p < 0.00001). Though most (85%) of the mothers/caretakers were advised on how to treat the children at home, only 9% were advised to bring their children back to the health facility if they saw any danger signs of malaria later on, a situation much worse than in 2000 (p = 0.00004). Most of the mothers/caretakers were however satisfied with the care provided to their children at the health facilities.

A total of 19,326 children under-five years of age were recorded to have been seen in the out patient department (OPD) by the five health facilities in the district within the last twelve months preceding the 2000 survey. Out of these, 53% were diagnosed as having clinical malaria; however, only 6.1% of the children had their diagnosis confirmed by microscopy. Many more children (30,248) were seen in 2003. Out of this, 56% were diagnosed as having clinical malaria. Though the percentage (14.4%) with laboratory confirmation of the malaria was low, it had significantly increased (p < 0.00001) compared with the levels in 2000.

The prevalence of anaemia (Hb < 10 g/dl) among children below five years of age seen at the OPD still reduced from a low of 1.9% in the 2000 survey to 0.6% in the 2003 survey (Table 7).

**Table 7:** Summary of clinical malaria burden indices in under five-year old children in the Kassena-Nankana district

	*Survey period*	
	
	2000	2003
	
*Clinical malaria burden indices*	(N = 2031) %	(N = 2208) %
Proportion of OPD cases with diagnosis of clinical malaria	53.0	56.0
Proportion of OPD malaria cases confirmed by microscopy	6.1	14.4
Proportion of OPD cases with diagnosis of anaemia	1.9	0.6
Proportion of IPD cases with diagnosis of clinical malaria	46.6	65.0
Proportion of IPD cases with diagnosis of anaemia	20.5	20.8
Anaemia case fatality	3.1	5.2
Malaria case fatality	3.5	4.0

The records of recently admitted under five year old children indicated that over 90% of these children were appropriately managed. Observational studies in the consultation rooms, indicated that the providers asked mothers/caretakers about the history of illness of their children; took necessary anthropometric and vital signs, and thorough physical examination. Where necessary, children were referred to the laboratory and mothers/caretakers were counselled.

The total number of inpatients under-five years of age recorded at the district hospital within the last 12 months prior to the 2000 survey was 2,031, with 46% diagnosed as having clinical malaria, and 20.5% anaemia. There were 103 deaths in the paediatric ward during the period with malaria case fatality rate of 3.5% and severe anaemia case fatality rate as 3.1%. The total number of inpatients under-five years within the last 12 months prior to the 2003 survey was 2,208, with 65% diagnosed as having clinical malaria, and 20.8% anaemia. There were 119 deaths in the paediatric ward during the period with malaria case fatality rate as 4% and severe anaemia case fatality rate as 5.2% (Table 7).

Available data from the NDSS indicate that all-cause mortality in children under five years of age in the KND was 164.5 per 1000 person-years in the year 2000 and 139.2 per 1000 in 2003.

## Discussion

The outcome variables compared in both surveys shows generally that care for patients with malaria (febrile illness) had improved over the years; an indication that significant progress was made by health providers in the strengthening of the health delivery system in the Kassena-Nankana district aimed at improving upon effective prevention and treatment strategies for children under five years of age and pregnant women. This has reflected in many more mothers/caretakers seeking treatment for their sick children within 24 hours of noticing that the child was unwell. During the period, many more mothers/caretakers got to know the more appropriate drugs for particular symptoms in children, for example giving paracetamol syrup to children with fever/hot body before sending the child to a health facility.

Though it is usually assumed that availability of health facilities will result to its utilisation by the community members, nearness to a health facility did not seem to influence the behaviour of mothers/caretakers as they sought care for their sick children. The improvement in care for children by their mothers/caretakers might be as a result of intensive health education being given to mothers at antenatal and child welfare clinics.

Though many more mothers/caretakers sent their sick children to health facilities over the study period, the role of chemical shops/drug shopkeepers in the health delivery system in the district is still highly significant. Most community members will send their health problems to the health facilities only when the drugs procured from these chemical shops/drug shopkeepers have failed to cure a particular ailment. The situation is very likely to improve as a result of the introduction of the district mutual health insurance scheme in the district which provides cheaper health care if one is able to pay the initial premium. For now, an intensive programme that focuses on educating and training of the chemical shop owners and drugs shopkeepers in the proper management of common ailments like acute malaria will help make full use of the early health-seeking behaviour of mothers/caretakers into providing safe, prompt and appropriate health care. Such training has become more critical with the introduction of the new anti-malarial drug policy in Ghana where the first line anti-malarial treatment is artesunate plus amodiaquine. The use of such training package for drug shopkeepers as reported by Marsh et al [7] in rural Kenya resulted in significant improvement in appropriate treatment using chloroquine over the counter.

Stocking of adequate anti-malarial drugs in the health facilities has to be ensured by health authorities as this will help build community confidence and not lead to instances whereby most patients who attended the hospital were provided with referral prescriptions to purchase their drugs from private chemical shops in the communities. Such instances tend to erode the important roles of the health facilities.

Even though almost every nursing mother had attended antenatal clinic (ANC) at least once during her most recent pregnancy, very few of them delivered at these facilities. According to D'Ambruoso et al [8] women expect humane, professional and courteous treatment from health professionals and a reasonable standard of physical environment. This may not be available at most health facilities. Similarly, accessibility factors like availability of transport, its cost and distance to the health facility may also contribute to the low delivery rate at health facilities [9]. This low level of utilization of maternity services could contribute to the high level of maternal and neonatal mortalities in this locality. Some significant improvement had taken place in the district by the time of the 2003 survey.

The availability of treatment bed nets to children and pregnant women has been encouraging as the situation improved significantly over the three-year period. This improvement could partly be due to the targeted sale [10] of subsidized treated nets for use by children under five years of age and pregnant women. This is a community in which a large-scale field trial of bed net was carried out [11] and so the people are fully aware of the benefits of ITNs.

Though the policy in Ghana supports anti-malarial chemoprophylaxis and now the use of intermittent preventive treatment during pregnancy, less than half of the respondents indicated using chemoprophylaxis during their most recent pregnancy, and of these, only 11% of the respondents in the 2000 survey used the indicated drugs appropriately (as prescribed). It is however, encouraging that the proportion of women using prophylaxis appropriately had increased significantly by the 2003 survey. At the moment, there is a district wide use of SP as chemoprophylaxis in the form of IPTp. This drug is more convenient to administer than chloroquine and is given at antenatal clinics. There is the need to intensify the health education to pregnant women by health staff in order to make the mothers appreciate the need for IPT. Proper malaria management practices during pregnancy have been shown to be beneficial to both mother and child in other communities [12,13].

It is also very encouraging that all the CHWs affirmed that they were able to treat uncomplicated cases of malaria presenting to them appropriately; and also identify danger signs of severe malaria leading to referrals to a higher level of care whenever necessary. Their role can be further enhanced by following up with programmes similar to that of Pagnoni et al [14] carried out in Burkina Faso where a reduction of cases of severe malaria seen at health facilities was observed as a result of CHWs providing pre-packed anti-malarials to mothers of sick children who had been educated to recognize malaria and treat appropriately and also refer promptly. Though the CHWs are interested in working with the health authorities without any remuneration, programmes will have to explore how to transform a completely voluntary service into some incentive-packaged service in order to make such a service sustainable.

The CBOs have indicated their preparedness to work with village health committees. Such groups could be facilitators in the dissemination of malaria control information such as the use of ITNs.

## Conclusion

The trend over this three-year period shows some improvement in malaria control activities in the district. This could mean that intensifying malaria control activities in this highly malarious area can make some positive impact on the debilitating effects of malaria, though the level of reduction over the three years may not be high enough to reduce the malaria burden by half by 2010 as indicated by Roll Back Malaria (RBM) in its malaria control target.

It is important to note that the current RBM tools can with slight modifications be used to monitor and evaluate malaria control activities in malaria endemic sites. One area that needs modification is the criteria for determining appropriateness of care and sample sizes required for each tool.

## Authors' contributions

OA was the principal investigator (PI) of the study. He led in the design and conduct of the study. He also participated in the analysis of the results and the preparation of this manuscript. EA was a co-investigator and study statistician. She participated in the design and conduct of the study. She also participated in the analysis of the results and the preparation of this manuscript. FA was a co-investigator and study co-ordinator. He contributed to the design of the study and co-ordinated it's conduct. He also participated in the analysis of the results and the preparation of this manuscript. MA was the study statistician. He contributed to the design of the study, results analysis and manuscript preparation. AH played a key role in the design and implementation of the study. TM was a co-PI. He was responsible for input on DHMT aspects of the work. EA played and advisory role in designing study. FB played an advisory role in designing and analysing data as well as write up. All authors read and approved the final manuscript.
